# Hypoxia-Ischemia or Excitotoxin-Induced Tissue Plasminogen Activator- Dependent Gelatinase Activation in Mice Neonate Brain Microvessels

**DOI:** 10.1371/journal.pone.0071263

**Published:** 2013-08-06

**Authors:** Priscilla L. Omouendze, Vincent J. Henry, Baptiste Porte, Nicolas Dupré, Peter Carmeliet, Bruno J. Gonzalez, Stéphane Marret, Philippe Leroux

**Affiliations:** 1 Haute-Normandie-INSERM ERI-28, Institute for Research and Innovation in Biomedicine of Rouen University, Rouen, France; 2 Laboratory of Angiogenesis and Neurovascular Link, Vesalius Research Center, VIB, Leuven, Belgium; 3 Department of Neonatal Paediatrics and Intensive Care, Rouen University Hospital, Rouen, France; Rutgers University, United States of America

## Abstract

Hypoxia-ischemia (HI) and excitotoxicity are validated causes of neonatal brain injuries and tissue plasminogen activator (t-PA) participates in the processes through proteolytic and receptor-mediated pathways. Brain microvascular endothelial cells from neonates in culture, contain and release more t-PA and gelatinases upon glutamate challenge than adult cells. We have studied t-PA to gelatinase (MMP-2 and MMP-9) activity links in HI and excitotoxicity lesion models in 5 day–old pups in wild type and in t-PA or its inhibitor (PAI-1) genes inactivated mice. Gelatinolytic activities were detected in SDS-PAGE zymograms and by *in situ* fluorescent DQ-gelatin microscopic zymographies. HI was achieved by unilateral carotid ligature followed by a 40 min hypoxia (8%O_2_). Excitotoxic lesions were produced by intra parenchymal cortical (i.c.) injections of 10 µg ibotenate (Ibo). Gel zymograms in WT cortex revealed progressive extinction of MMP-2 and MMP-9 activities near day 15 or day 8 respectively. MMP-2 expression was the same in all strains while MMP-9 activity was barely detectable in t-PA^−/−^ and enhanced in PAI-1^−/−^ mice. HI or Ibo produced activation of MMP-2 activities 6 hours post-insult, in cortices of WT mice but not in t-PA^−/−^ mice. In PAI-1^−/−^ mice, HI or vehicle i.c. injection increased MMP-2 and MMP-9 activities. *In situ* zymograms using DQ-gelatin revealed vessel associated gelatinolytic activity in lesioned areas in PAI-1^−/−^ and in WT mice. In WT brain slices incubated *ex vivo*, glutamate (200 µM) induced DQ-gelatin activation in vessels. The effect was not detected in t-PA^−/−^mice, but was restored by concomitant exposure to recombinant t-PA (20 µg/mL). In summary, neonatal brain lesion paradigms and *ex vivo* excitotoxic glutamate evoked t-PA-dependent gelatinases activation in vessels. Both MMP-2 and MMP-9 activities appeared t-PA-dependent. The data suggest that vascular directed protease inhibition may have neuroprotection potential against neonatal brain injuries.

## Introduction

Neonatal brain injuries are strongly associated to neonatal death or later disability in children. Besides a wide range of genetic defects, cerebral lesions acquired after hypoxia-ischemia and/or intracranial hemorrhage in the perinatal period predominates in at term and preterm infants, respectively. In Europe, the incidence of cerebral palsy vary in range from 1.5 to 2.5 per 1000 live births with little variation depending on state [1], [2]. Despite progress in the fields of obstetrics and neonatology, the rates of neurological disabilities in at term or preterm infants in the 1990s were comparable to those observed in the early 1960s [3]. The development of therapies is difficult because lesions occur while brain maturation is still ongoing in this period and maturity is different depending on brain regions. In fact, therapeutic interventions could also interfere with developmental processes in brain as well as periphery.

Animal models of neonatal brain injury have allowed partial understanding of lesion occurrence mechanisms. In the absence of tissue plasminogen activator (t-PA) the volume of excitotoxic lesions caused by intracerebral excitotoxin ibotenate injection was reduced in rodent neonates [4]. Under hypoxia-ischemia paradigm t-PA expression and activity are increased in hours after insult [5]. Deleterious potential of t-PA involved several mechanisms including inflammation cell activation in the neonatal brain [6]. The effect of t-PA partly depended on plasminogen activation since plasmin inhibitors reduced injuries in WT [5], [6] and had no effect in t-PA^−/−^ mice [6]. In adult ischemia degradation of vascular matrix proteins involve plasmin and matrix metalloproteinases (MMPs). Amongst MMPs, gelatinases (MMP-2, MMP-9) activity and expression are up-regulated after stroke [7]. MMP-9 appeared more likely the protease involved in endothelium basal lamina degradation and promote blood–brain barrier (BBB) disruption [8], [9], brain edema, infiltration of inflammation cells, and brain parenchyma damage [10], [11]. A relevant connection between neonatal stroke and MMP rely on the observation that i) increased MMP-9 and its inhibitor TIMP-1 levels were detected in neonates brain 24 h after hypoxia-ischemia [12], 2) MMP inhibitors are neuroprotective in neonatal stroke [13], 3) genetic alteration of collagen-IV correlates with porencephaly in neonates [14].

Recombinant t-PA thrombolytic therapy is performed in the management of stroke in adults [15], [16], but in addition to valuable fibrinolytic plasminogen-dependent activity, exogenous t-PA favors post-ichemia hemorrhage transformation, an effect limited by MMP inhibition in animal and in man [17]–[19]. In adult stroke patients undergoing t-PA thrombolysis and in animal models of ischemia, t-PA up-regulated plasma levels or brain MMP-9, while levels were reduced in t-PA knockout mice [20], [21]. Moreover, chemical activation of pro-MMP-9 zymogen by NO was reported in ischemia after t-PA dependent production [22].

In keeping with the variety of mechanisms involving t-PA and gelatinases, it is possible that the beneficial effect observed by inactivation of t-PA in neonatal animals results from gene repression and/or reduced activation of gelatinases. To test this hypothesis, we have used excitotoxic and hypoxo-ischemic experimental brain injuries in wild type and in genetically engineered (t-PA or PAI-1 knockout; t-PA^−/−^, PAI-1^−/−^) neonatal mice.

## Materials and Methods

### Animals

PAI-1 or t-PA knockout mice in C57Bl6/129 background (t-PA^−/−^ in 87.5/12.5% ratio and PAI-1^−/−^ in 75/25% ratio, respectively) were used and compared to respective wild type (WT) hybrids [23], [24]. Animals were housed in controlled temperature, humidity and day/night 12/12 hour cycle, with water and food *ad libitum*. A total number of 194 neonates have been used for the study.

The hypoxia-ischemia procedure derives from the procedure described in P7 rats [25] adapted to 5 day-old (P5) mice (day of birth counted P1). Right carotid ligation was performed under anesthesia with isoflurane (4% induction and 2% maintenance). Animals were returned for 1 h to their dam, and then submitted to 40 minutes hypoxia (O_2_/N_2_ ratio 8/92%). Temperature was maintained all along surgery by a heating carpet and then hypoxia was performed in a humidified and thermostated (33°C) device. Sham-operated animals were anesthetized, right carotid released but no ligation or hypoxia was done.

Excitotoxic insult by intra-cortical (i.c.) injection of 10 µg ibotenate (Sigma-Aldrich, Saint-Quentin-Fallavier, France) in phosphate buffered saline (PBS) was performed under 4% isoflurane anesthesia at P5 according to the protocol previously described [26]. Briefly, a 26-gauge mounted on a Hamilton syringe micro-dispenser device was inserted 3 mm below skin surface by an expert investigator approximately 3.5–3.7 mm from frontal border of olfactory bulb and 1,2 mm lateral to median line a posteriori confirmed with atlas [27]. Two 1 µl injections were performed accompanying device retraction at 10 seconds interval. Ten seconds were waited before needle complete retraction. Control animals received 2 µl vehicle instead of ibotenate.

### Ethics Statements

All procedures used were approved by the regional ethics committee for animal experimentation of Normandy (Agreement n° N/04-09-06/17 for intracerebral (i.c) injections and n° N/01-02-09/05/02-12 for hypoxic-ischemic procedure) and conducted in accordance with French law and recommendations of the National Institute for Health and Medical Research (INSERM) under supervision of authorized person (PL; Agrement, n° 76-A-16 reconducted n° B76-16, December 2012).

### Chemicals

Acetic acid, aprotinin, bovine serum albumin (BSA), calcium dichloride (CaCl2), Coomassie blue, ethanol, glycerol, glutamate, isopentane, ibotenic acid, isolectinB4-TRITC complex, PBS, sodium azide (NaN3), TritonX-100, Trizma Base, were from Sigma-Aldrich (St Quentin Fallavier, France). Dizocilpine (MK-801) was obtained from Tocris (R&D, Lille, France). SDS-PAGE Tris-glycine gel (containing 0.1% gelatin as gelatinases substrate), renaturation buffer, developing buffer and DQ-gelatin (used for in situ zymography) were from Invitrogen (Cergy Pontoise, France). DQ-gelatin consists of highly quenched, fluorescein-labeled gelatin, in which proteolytic digestion unmask green fluorescence visualized as an index of gelatinolytic activity. The gelatinase inhibitor SB-3CT was from Biomol (Lonza Sales LTD, Basel, Switzerland). Paraformaldehyde (PFA) was from Labonord (Templemars, France). Human recombinant t-PA (hrt-PA; Actilyse) was from Boerhinger Ingelheim (France).

### SDS-PAGE Gelatin Zymography

Gelatin zymograms revealed matrix metalloproteinase-2 (MMP-2) and MMP-9 activities in brain homogenates. The brains were quickly removed, separated into ipsilateral (injured) and contralateral cortices, frozen in liquid nitrogen and stored at -80°C for a maximum of 8 weeks. Frozen tissues were rapidly homogenized in ice-cold lysis buffer containing 50 mM Tris, 150 mM NaCl and 0.5% TritonX-100, pH = 7.4. After centrifugation, supernatants were collected, and protein concentrations were determined by Bradford assay (Sigma). Proteins were loaded (80 µg/well) and separated by electrophoresis. Then the gels were incubated in renaturation buffer for 30 min at room temperature (RT) and equilibrated in developing buffer for another 30 min. After transfer in fresh developing buffer, gels were incubated for 48 h at 37°C, stained in 0.5% Coomassie blue for 25 min and finally distained in 40% ethanol and 10% acetic acid. For determination of MMP-9 activities in lesion models 5–7 days incubations were necessary to detect activities. Light stripes on blue background indicate gelatin digestion in the gel by the gelatinases. Digital images of the gels were scanned using a Biorad device (Philadelphia, USA). Images were analyzed using ImageJ v.1.34 software (National Institutes of Health, Bethesda, MD). Surface and intensity of the bands were integrated with a color deconvolution algorithm, included in the ImageJ software and MMP activities expressed in arbitrary units. Two to 4 brains per age and genotype were used for ontogenic study. Zymograms from lesioned animals were performed at least 3 times.

### *In situ* Zymography

*In situ* gelatinolytic activity test was performed on 20 µm frozen brain sections. Frozen sections were thawed and incubated for 4 h at 37°C in 200 µL of 0.5 M Tris buffer (pH 7.6) containing 1.5 mM NaCl, 50 mM CaCl_2_ 2 mM NaN_3_ and 100 µg/mL DQ-gelatin in a humid chamber. After incubation sections were rinsed in PBS and fixed with PFA 4% on ice for 10 min, and then cover-slipped in PBS glycerol (50/50%). Sections were observed using an inverted Leica DMI6000 fluorescence microscope (Leica microsystems, Saint-Jorioz, France) and with a L5 and N2.1 filters for FITC and TRITC visualization, respectively. Image analysis was performed using Metamorph software (Roper Scientific, Evry, France).

### Lectino-histochemistry

Vessel labeling in the brain slices was achieved with isolectin-B4, a protein capable of scoring carbohydrates at the surface of vascular endothelial cells. The sections were put in incubation buffer (0.1 M PBS containing 0.3% TritonX-100 and Isolectin-B4-TRITC complex (1/50)) at 4°C overnight. After three rinses in PBS, the slices were mounted under coverslips in PBS/glycerol (50/50%).

### *Ex vivo* Slice Zymographies

Brain slices were obtained from WT or t-PA^−/−^ P5 mice. Animals were sacrificed by decapitation and their brains immediately placed into ice-cold artificial cerebrospinal fluid (aCSF; pH 7.4) containing (in mM): NaCl; 125, KCl; 3, CaCl_2_; 2, NaH_2_PO_4_; 1.2, MgSO_4_; 1.2, NaHCO_3_; 26, D-glucose; 10. Transverse slices (250 µm) were cut at 4°C using a vibrant microtome VT1000S (Leica-microsystems, Rueil-Malmaison, France). Symetric hemi-slices were separated in 300 µl aCSF wells and incubated in the presence of isolectin-B4-TRITC complex (1/50) for 30-min in a cell culture incubator (at 37°C in a 5% CO_2_ humidified atmosphere). After 3 washes with aCSF (5 min each) symmetric hemi-slices were incubated for 3 h with FITC-DQ-gelatin (1/25), in the presence of glutamate alone (200 µM), or associated with aprotinin (20 KIU/mL), The natural neurotransmitter glutamate was preferred to Ibotenic acid in this test as it is able in vitro to activate glutamate receptors, contrarily to in vivo paradigms in which uptake mechanisms prevent its toxicity. MK-801 (10 µM), SB-3CT (0.6 µM), hrt-PA (20 µg/mL) or combinations. Double labellings were observed under fluorescence microscope. Each experiment was reproduced 3 times in 2–3 slices per condition obtained from animals of each genotype.

### Statistical Analysis

Quantitation of gel zymogram was expressed as % of relative signal in control track (± standard error). Statistical comparisons were conducted using two way- Anova followed by Bonferronni test for intergroup comparisons and Student’*t* test. Differences with p<0.05 were considered significant. Statistical analyses are performed using the Graph-Pad Prism 4 software (San Diego, CA USA).

## Results

### Ontogeny of Brain Gelatinase during Development

**Figure 1:**
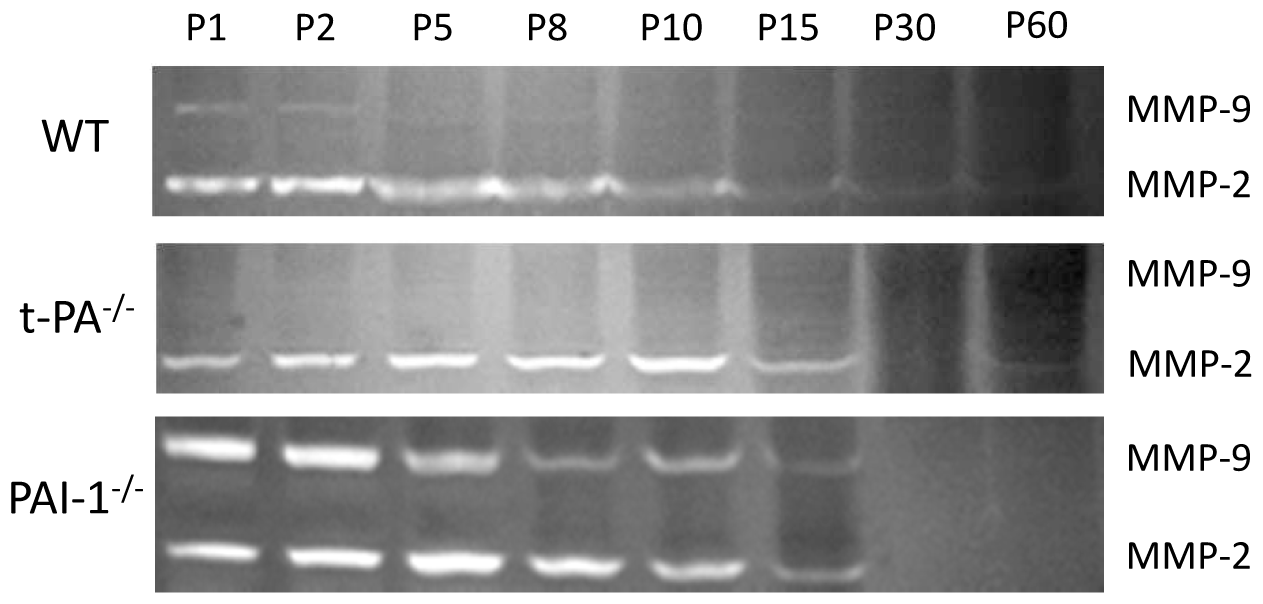
Ontogeny of gelatinase activity. Gelatin zymograms of mouse cortical homogenates prepared at postnatal days 1 (P1), to P60, in WT, t-PA^−/−^ or PAI-1^−/−^ animals. MMP-9 and MMP-2 activities are detected at 70 and 90 KDa respectively.

Gelatinolytic activity decreases with age in WT neonatal brain. MMP-2 activity progressively decreased from birth to become hardly detectable from P15 onwards. MMP-9 activity was lower than MMP-2 at all stages and became undetectable around P8 (Fig. 1A). Inactivation of t-PA or PAI-1 gene did not change the ontogenic expression patterns of MMP-2. Conversely MMP-9 that was hardly detected in WT was undetectable upon standard incubation in t-PA^−/−^ brain as early as P1. Reciprocally, in PAI-1^−/−^ neonates, high MMP-9 activity was detected, in the first two days, then progressively decreasing up to P15.

### Effect of hypoxia-ischemia on Gelatinase Zymograms

**Figure 2:**
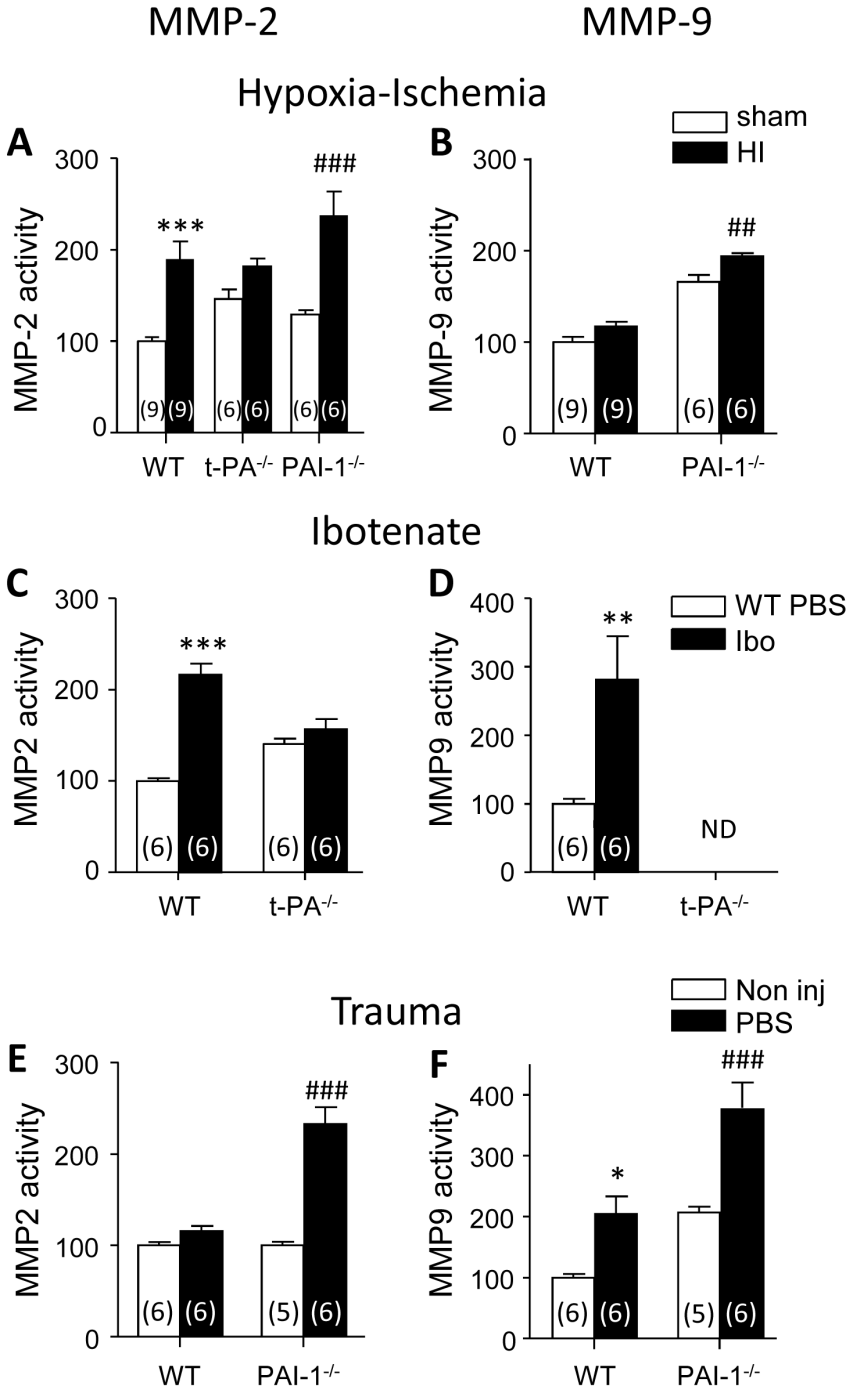
Quantification of gelatinase activities in cortical extracts 6 hours after insults in P5 mice. MMP-2 (A,C,E) and MMP-9 (B,D,F) gelatinolytic activities measured in hypoxia-ischemia (A,B), ibotenate excitotoxic paradigm (C,D) and PBS injection trauma (E,F). Data are expressed relative to the average densitometry in WT mice. * indicate significant difference with activity in respective controls, i.e. WT sham operated (A,B), PBS injected (C,D) or non-treated animals (E,F). *p<0.05; **p<0.01; ***p<0.001 vs WT controls,^ ##^p<0.001 and ^###^p<0.001 vs PAI-1^−/−^ controls. Numbers in parentheses indicate the number of animals used. ND; non detected upon standard incubation.

The effect of HI on gelatinase activities in gels was studied at P5 in WT, t-PA^−/−^ and PAI-1^−/−^ pups. Two-way Anova revealed significant effects of HI on MMP-2 activity, 6 hours after the end of the procedure (p<0.01), and an interaction of treatment and genotype (p<0.01). MMP-2 activity was increased by 90% in WT and 84% in PAI-1^−/−^ mice (p<0.001), while no significant increase was observed in t-PA^−/−^ mice (Fig. 2A). HI effect on MMP-9 activity studied by two-way Anova revealed a slight effect only in PAI-1^−/−^ mice (+17%; p<0.01) even if basal activity was high in this group (166% of WT control; p<0.001) (Fig. 2B). Very long term incubation of t-PA^−/−^ mice brain extracts allowed to revealing low MMP-9 activity at the same level in sham and HI exposed animals (not shown).

### Effect of Ibotenate Injections on Gelatinase Zymograms

Ibotenate effects were tested in WT and t-PA^−/−^ mice but not in PAI-1^−/−^ mice in which it produced huge hemorrhage (Fig. 2C–D). Control intracortical PBS injections induced microtrauma. Their effects were investigated in WT and in PAI-1^−/−^ mice in which t-PA activity was putatively dysregulated (Fig. 2E,F).

Ibotenate injection significantly increased the two gelatinase activities in WT mice cortex 6 hours after injection. MMP2 and MMP-9 activities were increased by 116% (p<0.001) and 181% (p<0.01), respectively). These effects were dependent on t-PA since MMP-2 activity was unaffected in t-PA^−/−^ mice (Fig. 2C) as well as MMP-9 revealed after prolonged incubation in these mice (not shown). Intracortical injection of PBS had no effect on MMP-2 activity in WT mice, but produced a 104% increase in MMP-9 activity (p<0.05)(Fig. 2E, F). In PAI-1^−/−^ mice the single injection of PBS i.c. induced 132% and 81% increases in MMP-2 (p<0.001) and MMP-9 (p<0.001) activities, compared to non-injected respective controls. The comparison of PBS i.c. effects in WT or PAI-1^−/−^ mice on MMP-9 activity did not reveal any interaction in two-way Anova confronting genotype and insult, even if basal activity in PAI-1^−/−^ mice were much higher (Fig. 2F). In fact the amplitude of MMP-9 activity increase observed in i.c. PBS injected mice was similar in both groups. Of note, 86.5% of i.c. PBS injected PAI-1^−/−^ pups exhibited parenchymal hemorrhage 24 h post-insult while no hemorrhage was observed in any other group.

### Effect of hypoxia-ischemia on Gelatinolytic Activity *in situ*

**Figure 3:**
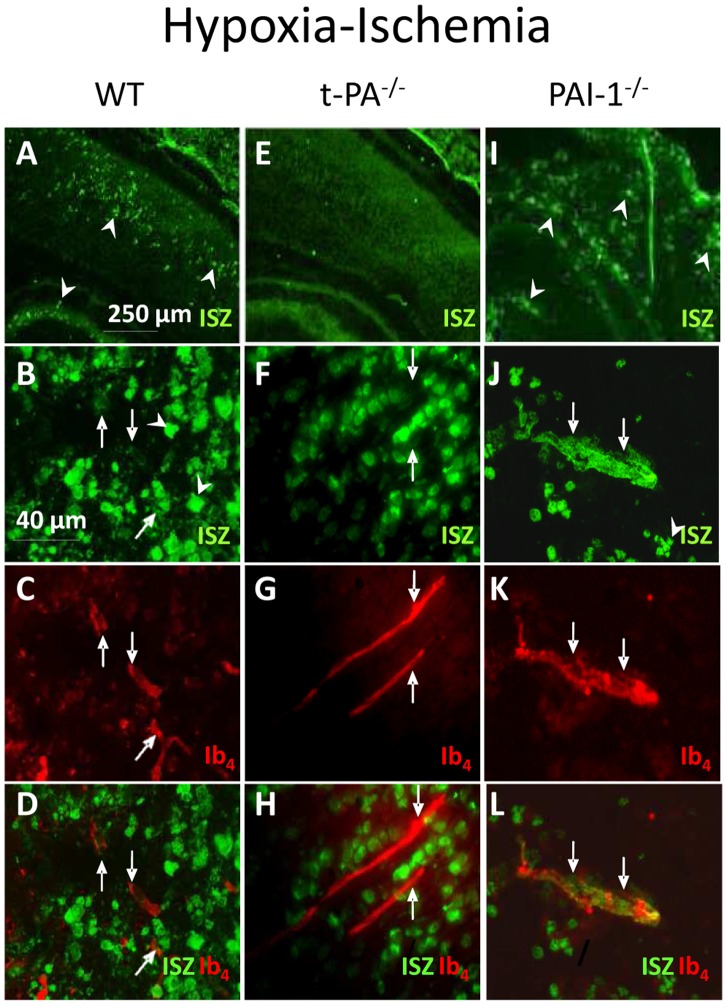
*In situ* gelatinolytic activity labeling 6 h after hypoxic-ischemic procedure. Gelatinolytic activity (green) and vessel labeling by IB4 (red) were obtained in WT (A–D), t-PA^−/−^ (E–H) and PAI-1^−/−^ (I–L) mice. Low magnification observation of gelatinolytic activity (A,E,I). Note gross tissue alteration in PAI-1^−/−^ and low level of fluorescence in t-PA^−/−^ mice sections. Higher magnification allows to visualizing micro-vascularization. Gelatinolytic activity is hardly detectable on vessels in WT (B–D), while it is undetectable in t-PA^−/−^ (F–H) and high in PAI-1^−/−^ (J–L) mice. Arrowheads point to spots of high activity; arrows point to microvessels. ISZ; *in situ* zymographic activity; Ib4; isolectin B4 vessels labeling.

*In situ* gelatinolytic visualization revealed spots of high activity in the somatosensory cortex and in the hippocampus in WT animals (Fig. 3A), while no sign of enhanced activity was notable in sections from t-PA^−/−^ mice (Fig. 3E). In WT mice, in situ gelatinolytic activity in vessels was present (Fig. 3 B–D), although it was hard to visualize due to much higher intensity in nervous cells nuclei located around (Fig. 3 F–H). Such an activity was not detected in t-PA^−/−^ mice (Fig. 3F–H). In PAI-1^−/−^ mice, the tissue was particularly altered with intense gelatinase activity spots (Fig. 3I). In these animals, gelatinase activity was also detected on the blood vessels labeled by isolectin B4 (Fig. 3J–L).

### Effect of Ibotenic Acid Injections on Gelatinolytic Activity *in situ*

**Figure 4:**
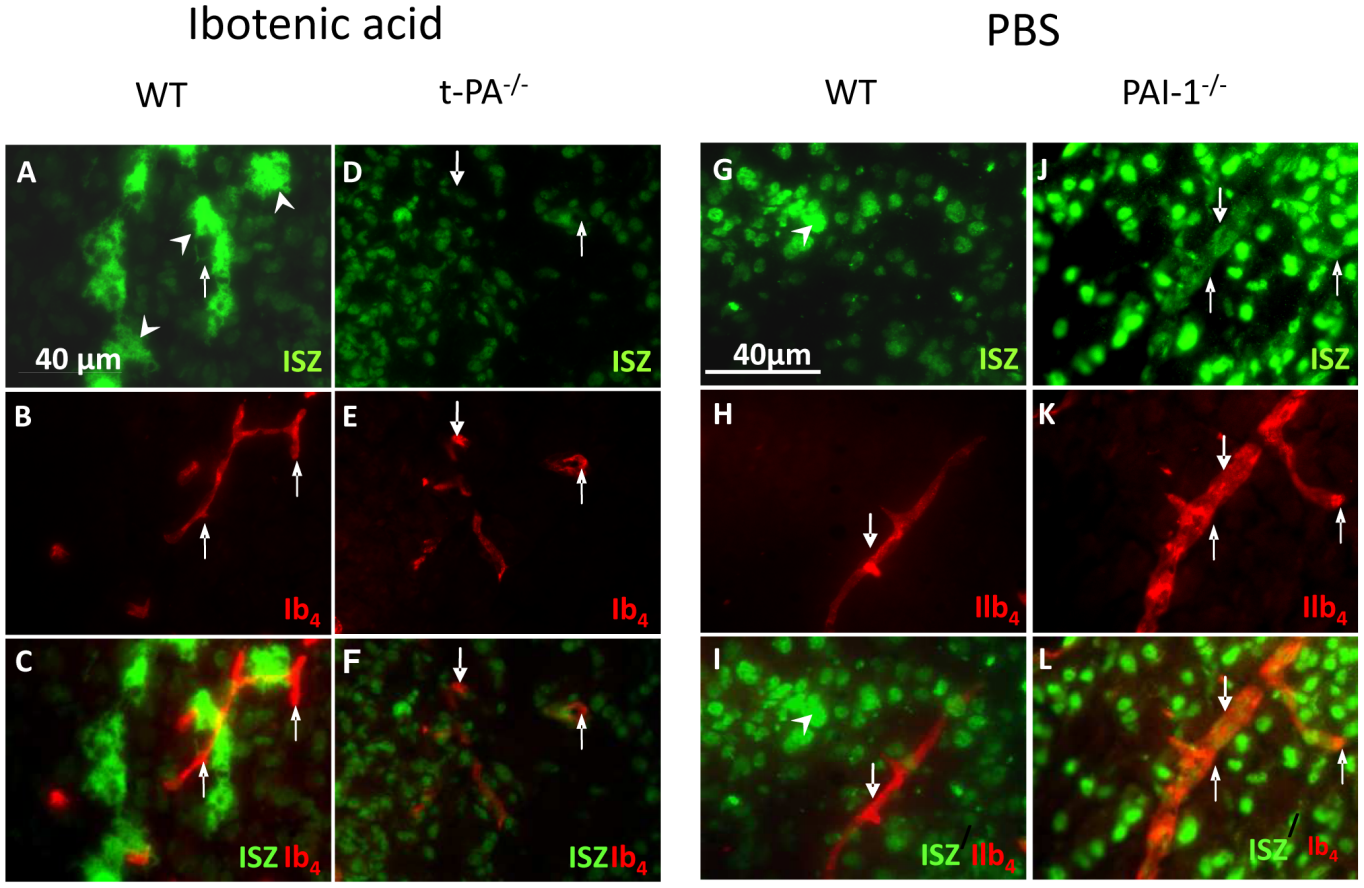
*In situ* gelatinolytic activity labeling 6 h after ibotenate injection. Ibotenate was injected in WT (A–C) and in t-PA^−/−^ (D–F) mice and PBS (control) was injected in WT (G–I) and PAI-1^−/−^ (J–L) mice. Ibotenate induced high gelatinolytic activity spots in WT but not in t-PA^−/−^ mice (A,D), but did not induce activity in microvessels (C,F). PBS controls in WT did not exhibit enhanced gelatinolytic ativity (G–I) but a strong intracellular activity together with vessel activity in PAI-1^−/−^ (J–L) mice. Arrowheads point to spots of high activity; arrows point to microvessels. ISZ; *in situ* zymographic activity; Ib4; isolectin B4 vessels labeling.

Ibotenic acid injections in WT mice resulted in intense gelatinolytic spots of activity largely over-spanning cells (Fig. 4A) compared to injection of PBS that did evoke sparse activity spots (Fig. 4G). The labeling of vessels with IB4 did not reveal gelatinolytic activity in vessels, although the very intense signal in spots may mask much lower activities in vessels (Fig. 4B,C). In t-PA^−/−^ mice ibotenate did not evoke gelatinolytic activity in nerve cells (Fig. 4D) nor in vessels (Fig. 4E,F). In PAI-1^−/−^ animals, the sole injection of PBS clearly evoked gelatinolytic activity in vessels (Fig. 4J–L). No such effect was observed in WT mice (Fig. 4H–I).

### Long Term Effects

The effects of hypoxia-ischemia, ibotenic acid or PBS injection at P5 in the different types of mice were examined 5 days after insults. 5 days after HI, MMP-2 activity in gel had returned to basal activity in all animals. No significant difference was observed in the different groups (not shown).

**Figure 5:**
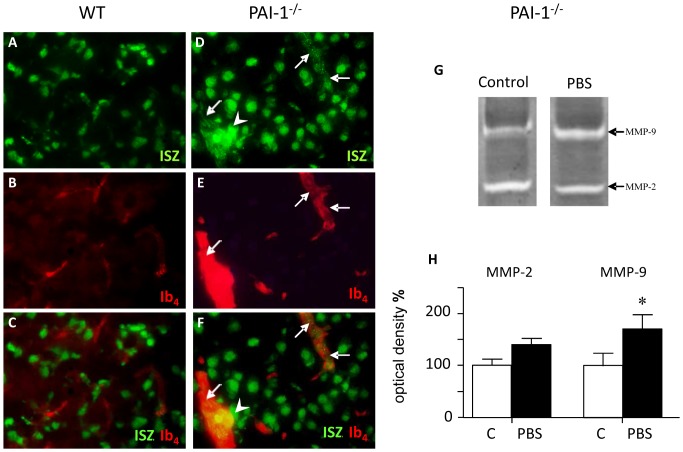
*In situ* and *in vitro* gelatinolytic activity 5 days after PBS injection in WT and PAI-1^−/−^ mice. Effect of PBS intracortical injection in WT (A–C) or PAI-1^−/−^ (D–F) 5 days post-insult. Gelatinolytic activity remained elevated in PAI-1^−/−^ mice in cell nuclei, in extracellular spots and in vessels. (**G**) Gel zymogram of gelatinase activity in PAI-1^−/−^ mouse cortex 5 days after PBS injection at P5 and non-injected control. (**H**) Quantification of MMP-2 and MMP-9 activities in gels. Arrowheads point to spots of high activity; arrows point to microvessels. Numbers in parentheses indicate the number of animals used. *p<0.05 compared to corresponding controls, according to Student’*t* test.

In WT mice, the injection of PBS did not produce significant hemorrhage and most of time any tissue damage detectable in cresyl violet staining 5 days post-insult [4], [28]. Conversely, in PAI-1^−/−^ pups, the injection trauma produced delayed hemorrhage (from 24 hours post injection onwards) with recurrences for several days (unpublished observation). In these animals, gelatinolytic activity in spots and in vessels were detected *in situ* 5 days after trauma (Fig. 5D–F), while it had returned to basal level in WT mice (Fig. 5A–C). In PAI-1^−/−^ mice, global MMP-9 activity in gel was enhanced (+71%, p = 0.012, according to Student’ *t* test), while the modest increase in MMP-2 activity (+41%) did not reach statistical significance (Fig. 5G,H). In all other contexts; HI or ibotenate injections in WT or t-PA^−/−^ mice, no MMP-9 activation was detected after 5 days (not shown).

### *Ex vivo* Gelatinase Assay in Slices

**Figure 6:**
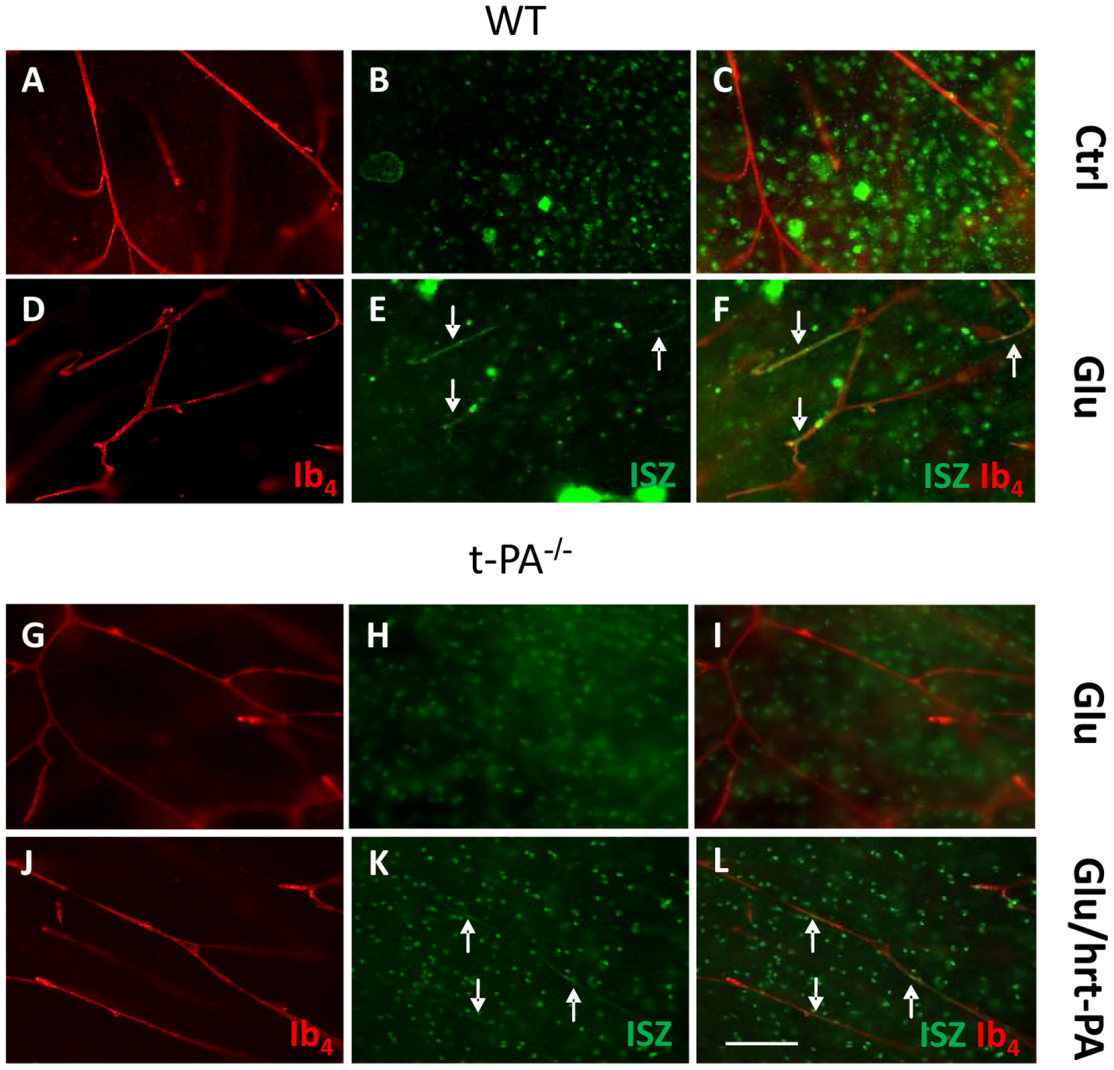
Vascular gelatinolytic activity labeling in 5 day-old mice brain sections exposed to glutamate and hrt-PA. Gelatinolytic activity (green) and vessel labeling by IB4 (red) were obtained in WT (A–F) or t-PA^−/−^ (G–L) mouse brain sections (250 µm thick) after 3 hours *ex vivo* exposure to control medium (A–C), glutamate 200 µM alone (D–I) or in association with 20 µg/mL hrt-PA (J–L). Arrows point out gelatinolytic activity in vessels. Bar = 200 µm.

*Ex vivo* WT brain slice incubations in aCSF allowed to reveal gelatinase activity in cells but not in vessels (Fig. 6A–C). Glutamate (200 µM) exposure induced vascular gelatinase activities (Fig. 6D–F). The effect of glutamate was reversed by co-incubation with the NMDA receptor blocker MK801, the plasmin inhibitor aprotinin or the gelatinase inhibitor SB-3CT (not shown).

In t-PA^−/−^ mouse slices, glutamate (200 µM) failed to induce gelatinase activation in vessels (Fig. 6G–I) while combination of glutamate with hrt-PA (20 µg/mL) evoked gelatinase activation in vessels (Fig. 6J–L). hrt-PA used alone was also able to induce DG-gelatin cleavage in some microvessels (not shown).

## Discussion

Ontogenic study shows that gelatinase activity decreases during brain development to undetectable levels after the first two weeks postnatal. MMP-2 profile obtained in C57Bl6/129 hybrids is very similar to the profile described in C57Bl6 mice. Basal MMP-9 activity disappeared earlier, detected up to P5 in this strain although it was not observed postnatally in Swiss mice [29]. Few studies described roles for MMP-2 and MMP-9 in neurogenesis, neuron migration and oligodendrogliogenesis during brain development [30]. In rats, MMP2/9 transcripts, proteins and activities decreased along with post-natal cerebellar histogenesis [31]. The decline observed in the present study, similar for MMP-2 activity in WT, t-PA^−/−^ and PAI-1^−/−^ neonates, appeared t-PA/PAI-1 dependent for MMP-9 activity, evocative of a positive relationship with t-PA levels; null in t-PA^−/−^, regulated in WT and likely elevated in PAI-1^−/−^ animals. This implies that t-PA is a positive regulator of basal MMP-9 activity in postnatal cortical development in mice.

The effects of t-PA may arise by plasmin activation which would in turn cleave pro-MMP-9 zymogen [32] or by MMP-9 expression evoked by t-PA through LRP-1 receptor activation [33]. Recently the action of the t-PA-PAI-1 complex was shown an inducer of a MMP cascade through LRP-1 activation in brain trauma resulting in vascular permeability and oedema [34]. Indeed endogenous t-PA activity at LRP1 is associated with weakening of perivascular unit in ischemia [35] and may lead to hemorrhage transformation of infarct post-ischemia [17]. The potentiating effect of t-PA in NMDA glutamate receptor efficiency may also result in gelatinase activity by enhanced nitric oxide (NO) synthesis and release [22], [36]. In fact NO is able to promote MMP-9 activation by nitrosylation of cysteine in the enzyme pro-domain thus unmasking the catalytic site [37]–[39]. In rat neonates, HI also provokes acute induction of plasminogen activators (t-PA and urokinase) that did not occur in adults [5].

It is generally accepted that MMP activities increase in pathological conditions but unraveling the cellular sources of gelatinases remains complex [10], [13]. *In situ* gelatinolytic studies show that both intra- and extracellular activities were elicited under noxious conditions. The actual selectivity of DQ-gelatin does not allow to identifying the MMP subtypes activities detected in nuclei. However it is likely that they participate in neuron survival or death fate, rather than in vascular impairment [40]–[42]. The extracellular spots detected in HI in WT and PAI-1^−/−^ or ibotenate injected mice may be due to MMP-9 microglial release as previously described [43]. In vessels endothelial and pericyteal locations could occur, but *in situ* zymograms images are also compatible with vascular basal lamina matrix-linked activity resulting from cleavage of resident pro-enzymes. The present observations that in WT mice HI elicited MMP-2 but not MMP-9 activation in crude extracts, suggest a t-PA to MMP-2 activation link mechanism that may not require MMP-9 activation. Although a t-PA-MMP-9 activation link is often reported in the literature, a t-PA-MMP2 link is not referred to [44]. However, the role of MMP-2 in ischemia was reported in adult models, in which its rapid elevation mediated BBB impairments [44], [45]. In human neonates, such an effect on basal lamina impairment and vessel weakening is likely since MMP-2 activity is localized in developing vessels [46].

MMP-9 has been associated to blood brain barrier disruption and to vascular defect in adults [47], [48], and likely in neonates [43]. We have previously reported that cultured neonatal microvascular endothelial cells secreted higher amounts of t-PA and gelatinases than adult cells in basal condition or under glutamate challenge, and that glutamate induced Evans blue extravasation in isolated microvessels more efficiently in neonatal than in adult preparations [49], [50]. Whether ibotenate did not produced vascular gelatinase activation in WT mice in vivo while glutamate evoked activity in vitro may be due to rapid clearance of ibotenat in vivo, differences in receptors recruited or time course of vascular response. Anyway, the present data further demonstrate a functional link between high glutamate, t-PA and vascular bed MMP activation. The effect is mimicked by direct t-PA application, while genetic inactivation (t-PA^−/−^) or pharmacological plasmin blockade (aprotinin) disrupted the functional chain. Reciprocally, the inactivation of PAI-1 that allowed unrestrained t-PA activity, facilitated the visualization of diffuse gelatinase activity in vessels, in line with extracellular activity. In these animals the sole PBS injection trauma evoked lasting MMP activity at least 5 days post-insult coincident with intracerebral bleeding in the majority of pups (unpublished data). This observation consolidates the hypothesis that a t-PA to MMP link in neonates may be involved in intraparenchymal/intraventricular bleeding which is highly incident in extreme preterms. In these infants, blood t-PA increased in the first postnatal days [51] and the elevated PAI-1 to t-PA ratio is evocative of vascular risk [34], [52]. Indeed, in neonatal encephalopathy the levels of blood MMP-9 is about 10-fold higher than in non-encephalopathic controls [12].

### Conclusion

The study shows that t-PA is involved in basal expression of MMP-9 during development and both MMP-2 and MMP-9 activation under HI and excitotoxicity lesion paradigms in neonates. A glutamate-evoked t-PA-dependent highly sensitive gelatinase activation in vessels likely sustains a pro-hemorrhage risk in neonates.

## References

[1] Himmelmann K, Hagberg G, Uvebrant P (2010) The changing panorama of cerebral palsy in Sweden. X. Prevalence and origin in the birth-year period 1999–2002. Acta Paediatr. 99: 1337–1343. 10.1111/j.1651-2227.2010.01819.x 20377538

[2] Paneth N, Hong T, Korzeniewski S (2006) The descriptive epidemiology of cerebral palsy. Clin Perinatol. 33: 251–267. 10.1016/j.clp.2006.03.011 16765723

[3] Himmelmann K, Hagberg G, Beckung E, Hagberg B, Uvebrant P (2005) The changing panorama of cerebral palsy in Sweden. IX. Prevalence and origin in the birth-year period 1995–1998. Acta Paediatr. 94: 287–294. 10.1111/j.1651-2227.2005.tb03071.x 16028646

[4] Hennebert O, Marret S, Carmeliet P, Gressens P, Laquerriere A, et al (2004) Role of tissue-derived plasminogen activator (t-PA) in an excitotoxic mouse model of neonatal white matter lesions. J Neuropathol Exp Neurol. 63: 53–63. 10.1093/jnen/63.1.53 14748561

[5] Adhami F, Yu D, Yin W, Schloemer A, Burns KA, et al (2008) Deleterious effects of plasminogen activators in neonatal cerebral hypoxia-ischemia. Am J Pathol. 172: 1704–1716. 10.2353/ajpath.2008.070979 PMC2408429 18467699

[6] Leroux P, Hennebert O, Legros H, Laudenbach V, Carmeliet P, et al (2007) Role of tissue-plasminogen activator (t-PA) in a mouse model of neonatal white matter lesions: Interaction with plasmin inhibitors and anti-inflammatory drugs. Neuroscience. 146: 670–678. 10.1016/j.neuroscience.2007.01.029 17321054

[7] Candelario-Jalil E, Yang Y, Rosenberg GA (2009) Diverse roles of matrix metalloproteinases and tissue inhibitors of metalloproteinases in neuroinflammation and cerebral ischemia. Neuroscience. 158: 983–994. 10.1016/j.neuroscience.2008.06.025 PMC3584171 18621108

[8] Bauer AT, Burgers HF, Rabie T, Marti HH (2010) Matrix metalloproteinase-9 mediates hypoxia-induced vascular leakage in the brain via tight junction rearrangement. J Cereb Blood Flow Metab. 30: 837–848. 10.1038/jcbfm.2009.248 PMC2949161 19997118

[9] Fukuda S, Fini CA, Mabuchi T, Koziol JA, Eggleston LL Jr, et al (2004) Focal cerebral ischemia induces active proteases that degrade microvascular matrix. Stroke. 35: 998–1004. 10.1161/01.STR.0000119383.76447.05 PMC2979008 15001799

[10] Morancho A, Rosell A, Garcia-Bonilla L, Montaner J (2010) Metalloproteinase and stroke infarct size: role for anti-inflammatory treatment? Ann N Y Acad Sci. 1207: 123–133. 10.1111/j.1749-6632.2010.05734.x 20955435

[11] Rosell A, Cuadrado E, Ortega-Aznar A, Hernandez-Guillamon M, Lo EH, et al (2008) MMP-9-positive neutrophil infiltration is associated to blood-brain barrier breakdown and basal lamina type IV collagen degradation during hemorrhagic transformation after human ischemic stroke. Stroke. 39: 1121–1126. 10.1161/STROKEAHA.107.500868 18323498

[12] Bednarek N, Svedin P, Garnotel R, Favrais G, Loron G, et al (2012) Increased MMP-9 and TIMP-1 in mouse neonatal brain and plasma and in human neonatal plasma after hypoxia-ischemia: a potential marker of neonatal encephalopathy. Pediatr Res. 71: 63–70. 10.1038/pr.2011.3 22289852

[13] Chen W, Hartman R, Ayer R, Marcantonio S, Kamper J, et al (2009) Matrix metalloproteinases inhibition provides neuroprotection against hypoxia-ischemia in the developing brain. J Neurochem. 111: 726–736. 10.1111/j.1471-4159.2009.06362.x 19712057

[14] Gould DB, Phalan FC, Breedveld GJ, van Mil SE, Smith RS, et al (2005) Mutations in Col4a1 cause perinatal cerebral hemorrhage and porencephaly. Science. 308: 1167–1171. 10.1126/science.1109418 15905400

[15] Buchan AM, Barber PA, Newcommon N, Karbalai HG, Demchuk AM, et al (2000) Effectiveness of t-PA in acute ischemic stroke: outcome relates to appropriateness. Neurology. 54: 679–684. 10.1212/wnl.54.3.679 10680803

[16] Christoforidis GA, Slivka AP, Karakasis C, Mohammad Y, Avutu B, et al (2010) Hemorrhage rates and outcomes when using up to 100 mg intra-arterial t-PA for thrombolysis in acute ischemic stroke. Interv Neuroradiol. 16: 297–305. 10.1177/159101991001600312 PMC3277989 20977864

[17] Tang J, Li YJ, Li Q, Mu J, Yang DY, et al (2010) Endogenous tissue plasminogen activator increases hemorrhagic transformation induced by heparin after ischemia reperfusion in rat brains. Neurol Res. 32: 541–546. 10.1179/174313209X414560 19309545

[18] Montaner J, Molina CA, Monasterio J, Abilleira S, Arenillas JF, et al (2003) Matrix metalloproteinase-9 pretreatment level predicts intracranial hemorrhagic complications after thrombolysis in human stroke. Circulation. 107: 598–603. 10.1161/01.cir.0000046451.38849.90 12566373

[19] Aoki T, Sumii T, Mori T, Wang X, Lo EH (2002) Blood-brain barrier disruption and matrix metalloproteinase-9 expression during reperfusion injury: mechanical versus embolic focal ischemia in spontaneously hypertensive rats. Stroke. 33: 2711–2717. 10.1161/01.str.0000033932.34467.97 12411666

[20] Tsuji K, Aoki T, Tejima E, Arai K, Lee SR, et al (2005) Tissue plasminogen activator promotes matrix metalloproteinase-9 upregulation after focal cerebral ischemia. Stroke. 36: 1954–1959. 10.1161/01.STR.0000177517.01203.eb 16051896

[21] Ning M, Furie KL, Koroshetz WJ, Lee H, Barron M, et al (2006) Association between tPA therapy and raised early matrix metalloproteinase-9 in acute stroke. Neurology. 66: 1550–1555. 10.1212/01.wnl.0000216133.98416.b4 16717217

[22] Parathath SR, Parathath S, Tsirka SE (2006) Nitric oxide mediates neurodegeneration and breakdown of the blood-brain barrier in tPA-dependent excitotoxic injury in mice. J Cell Sci. 119: 339–349. 10.1242/jcs.02734 16410551

[23] Carmeliet P, Kieckens L, Schoonjans L, Ream B, Van Nuffelen A, et al (1993) Plasminogen activator inhibitor-1 deficient mice, I: generation by homologous recombination and characterization. J Clin Invest. 92: 2746–2755. 10.1172/JCI116892 PMC288473 8254028

[24] Carmeliet P, Schoonjans L, Kieckens L, Ream B, Degen J, et al (1994) Physiological consequences of loss of plasminogen activator gene function in mice. Nature. 368: 419–424. 10.1038/368419a0 8133887

[25] Rice JE, 3rd, Vannucci RC, Brierley JB (1981) The influence of immaturity on hypoxic-ischemic brain damage in the rat. Ann Neurol. 9: 131–141. 10.1002/ana.410090206 7235629

[26] Marret S, Mukendi R, Gadisseux JF, Gressens P, Evrard P (1995) Effect of ibotenate on brain development : an excitotoxic mouse model of microgyria and posthypoxic-like lesions. J Neuropathol Exp Neurol. 54: 358–370. 10.1097/00005072-199505000-00009 7745435

[27] Paxinos G, Halliday G, Watson CS, Koutcherov Y, Wang HQ (2007) *Atlas of the developing mouse brain at E17.5, P0, and P6*. 1rst ed., Burlington: Academic Press. 374.

[28] Hennebert O, Laudenbach V, Laquerrière A, Verney C, Carmeliet P, et al (2005) Ontogenic study of the influence of tissue plasminogen activator (t-PA) in neonatal excitotoxic brain insult and the subsequent microglia/macrophage activation. Neuroscience. 130: 697–712. 10.1016/j.neuroscience.2004.09.049 15590153

[29] Bednarek N, Clement Y, Lelievre V, Olivier P, Loron G, et al (2009) Ontogeny of MMPs and TIMPs in the murine neocortex. Pediatr Res. 65: 296–300. 10.1203/PDR.0b013e3181973aee 19092727

[30] Agrawal SM, Lau L, Yong VW (2008) MMPs in the central nervous system: where the good guys go bad. Semin Cell Dev Biol. 19: 42–51. 10.1016/j.semcdb.2007.06.003 17646116

[31] Ayoub AE, Cai TQ, Kaplan RA, Luo J (2005) Developmental expression of matrix metalloproteinases 2 and 9 and their potential role in the histogenesis of the cerebellar cortex. J Comp Neurol. 481: 403–415. 10.1002/cne.20375 15593342

[32] Zhao BQ, Ikeda Y, Ihara H, Urano T, Fan W, et al (2004) Essential role of endogenous tissue plasminogen activator through matrix metalloproteinase 9 induction and expression on heparin-produced cerebral hemorrhage after cerebral ischemia in mice. Blood. 103: 2610–2616. 10.1182/blood-2003-03-0835 14630814

[33] Zhang C, An J, Haile WB, Echeverry R, Strickland DK, et al (2009) Microglial low-density lipoprotein receptor-related protein 1 mediates the effect of tissue-type plasminogen activator on matrix metalloproteinase-9 activity in the ischemic brain. J Cereb Blood Flow Metab. 29: 1946–1954. 10.1038/jcbfm.2009.174 19672275

[34] Sashindranath M, Sales E, Daglas M, Freeman R, Samson AL, et al (2012) The tissue-type plasminogen activator-plasminogen activator inhibitor 1 complex promotes neurovascular injury in brain trauma: evidence from mice and humans. Brain. 135: 3251–3264. 10.1093/brain/aws178 PMC3501968 22822039

[35] Polavarapu R, Gongora MC, Yi H, Ranganthan S, Lawrence DA, et al (2007) Tissue-type plasminogen activator-mediated shedding of astrocytic low-density lipoprotein receptor-related protein increases the permeability of the neurovascular unit. Blood. 109: 3270–3278. 10.1182/blood-2006-08-043125 PMC1852247 17170123

[36] Nicole O, Docagne F, Ali C, Margaill I, Carmeliet P, et al (2001) The proteolytic activity of tissue-plasminogen activator enhances NMDA receptor-mediated signaling. Nat Med. 7: 59–64. 10.1038/83358 11135617

[37] Chung KK, Dawson VL, Dawson TM (2005) S-nitrosylation in Parkinson’s disease and related neurodegenerative disorders. Methods Enzymol. 396: 139–150. 10.1016/S0076-6879(05)96014-X 16291229

[38] Gu Z, Kaul M, Yan B, Kridel SJ, Cui J, et al (2002) S-nitrosylation of matrix metalloproteinases: signaling pathway to neuronal cell death. Science. 297: 1186–1190. 10.1126/science.1073634 12183632

[39] Manabe S, Gu Z, Lipton SA (2005) Activation of matrix metalloproteinase-9 via neuronal nitric oxide synthase contributes to NMDA-induced retinal ganglion cell death. Invest Ophthalmol Vis Sci. 46: 4747–4753. 10.1167/iovs.05-0128 16303975

[40] Lee SR, Tsuji K, Lee SR, Lo EH (2004) Role of matrix metalloproteinases in delayed neuronal damage after transient global cerebral ischemia. J Neurosci. 24: 671–678. 10.1523/JNEUROSCI.4243-03.2004 PMC6729252 14736853

[41] Yang Y, Candelario-Jalil E, Thompson JF, Cuadrado E, Estrada EY, et al (2010) Increased intranuclear matrix metalloproteinase activity in neurons interferes with oxidative DNA repair in focal cerebral ischemia. J Neurochem. 112: 134–149. 10.1111/j.1471-4159.2009.06433.x PMC4950937 19840223

[42] Hill JW, Poddar R, Thompson JF, Rosenberg GA, Yang Y (2012) Intranuclear matrix metalloproteinases promote DNA damage and apoptosis induced by oxygen-glucose deprivation in neurons. Neuroscience. 220: 277–290. 10.1016/j.neuroscience.2012.06.019 PMC4546359 22710064

[43] Svedin P, Hagberg H, Savman K, Zhu C, Mallard C (2007) Matrix metalloproteinase-9 gene knock-out protects the immature brain after cerebral hypoxia-ischemia. J Neurosci. 27: 1511–1518. 10.1523/JNEUROSCI.4391-06.2007 PMC6673738 17301159

[44] Chang DI, Hosomi N, Lucero J, Heo JH, Abumiya T, et al (2003) Activation systems for latent matrix metalloproteinase-2 are upregulated immediately after focal cerebral ischemia. J Cereb Blood Flow Metab. 23: 1408–1419. 10.1097/01.WCB.0000091765.61714.30 14663336

[45] Liu J, Jin X, Liu KJ, Liu W (2012) Matrix metalloproteinase-2-mediated occludin degradation and caveolin-1-mediated claudin-5 redistribution contribute to blood-brain barrier damage in early ischemic stroke stage. J Neurosci. 32: 3044–3057. 10.1523/JNEUROSCI.6409-11.2012 PMC3339570 22378877

[46] Girolamo F, Virgintino D, Errede M, Capobianco C, Bernardini N, et al (2004) Involvement of metalloprotease-2 in the development of human brain microvessels. Histochem Cell Biol. 122: 261–270. 10.1007/s00418-004-0705-x 15375663

[47] Mantuano E, Inoue G, Li X, Takahashi K, Gaultier A, et al (2008) The hemopexin domain of matrix metalloproteinase-9 activates cell signaling and promotes migration of schwann cells by binding to low-density lipoprotein receptor-related protein. J Neurosci. 28: 11571–11582. 10.1523/JNEUROSCI.3053-08.2008 PMC3837707 18987193

[48] Yang Y, Hill JW, Rosenberg GA (2011) Multiple roles of metalloproteinases in neurological disorders. Prog Mol Biol Transl Sci. 99: 241–263. 10.1016/B978-0-12-385504-6.00006-3 21238938

[49] Legros H, Launay S, Roussel BD, Marcou-Labarre A, Calbo S, et al (2009) Newborn- and adult-derived brain microvascular endothelial cells show age-related differences in phenotype and glutamate-evoked protease release. J Cereb Blood Flow Metab. 29: 1146–1158. 10.1038/jcbfm.2009.39 19367295

[50] Henry VJ, Lecointre M, Laudenbach V, Ali C, Macrez R, et al (2013) High t-PA release by neonate brain microvascular endothelial cells under glutamate exposure affects neuronal fate. Neurobiol Dis. 50: 201–208. 10.1016/j.nbd.2012.10.020 23103420

[51] Sentilhes L, Leroux P, Radi S, Ricbourg-Schneider A, Laudenbach V, et al (2011) Influence of Gestational-age on Fibrinolysis from Birth to Postnatal Day 10. J. Pediatrics. 158: 377–382. 10.1016/j.jpeds.2010.08.033 20889163

[52] Leroux P, Marret S (2013) PAI-1/t-PA ratio in cord blood: a potential index of brain hemorrhage risk in extreme preterms. Arch Dis Child Fetal Neonatal Ed. doi: –10.1136/archdischild-2012–303603 10.1136/archdischild-2012-303603 23475899

